# Comparison of Medicaid financing for birth centers: a nine-state policy analysis

**DOI:** 10.3389/frhs.2025.1569462

**Published:** 2025-07-08

**Authors:** Rebecca H. Ofrane, Leslie Kantor, Julie Blumenfeld, Slawa Rokicki

**Affiliations:** ^1^ Department of Urban-Global Health, Rutgers University School of Public Health, Piscataway, NJ, United States; ^2^ Division of Advanced Nursing Practice, Rutgers University School of Nursing, Newark, NJ, United States

**Keywords:** birth centers, Medicaid, health policy, midwives, health economics, maternal health

## Abstract

**Background:**

The United States continues to face poor maternal health outcomes, and freestanding birth centers are a safe alternative to hospitals, offering midwifery care for low-risk birthing people. Not all birth centers accept Medicaid patients, however, and among those that do, low Medicaid facility reimbursement rates are a barrier to birth center operations and sustainability. This limits access especially for low-risk birthing people of color who may perceive traditional hospital care to be unsafe or culturally unsupportive.

**Methods:**

This cross-sectional policy analysis explored variation in U.S. Medicaid reimbursement rates for birth center facility fees. State similarity methods were used to match comparable states to New Jersey due to the state's evolving policy environment, resulting in a nine-state sample for the policy analysis.

**Results:**

Of this sample, six had published Medicaid rates for the birth center facility fee, with wide variation among the states, New Jersey's being the lowest and Massachusetts the highest, at four-and-a-half times New Jersey's rate. Significant variation in reimbursement rates was also identified when transfer to a hospital occurs.

**Conclusions:**

The findings suggest the importance of Medicaid reimbursement rates for birth centers as a policy pathway to improving access to this under-utilized care setting.

## Introduction

Birth centers are midwifery-led alternatives to hospitals for birth, typically centered around the experience of the birthing person and family, and are available for individuals with healthy, uncomplicated pregnancies and births (1). Yet hospitals remain the default birth setting in the U.S., sustaining the medicalization of birth, with the overuse of perinatal interventions described by the National Academies of Science and Medicine as “too much, too soon” (1). A systematic review of studies assessing maternal health outcomes for people beginning intrapartum (labor) care in birth centers vs. hospitals found significantly improved outcomes for those beginning care in birth centers, regardless of hospital transfer status or racial/ethnic group (2). Experience and satisfaction with perinatal care is also a critical indicator of outcomes, and birth centers offer promising improvements. Among Black and Hispanic birthing people, roughly 30% report mistreatment during perinatal care broadly, and almost 40% report discrimination during maternity care (3). Yet superior quality experience is evidenced among birth center users (2). Birth center births have also been found to cost less than hospital births, driven primarily by reduced interventions, cesarean rates, shorter facility stays, and fewer emergency department visits during the first year after birth (1, 4).

Nationally, there were 415 freestanding birth centers in 2024, but only 0.64% of births were in a freestanding birth center (5). Yet Declercq and colleagues' survey of mothers who had given birth in a hospital shows that 64% expressed an interest in a birth center for a future birth, with one quarter saying they “definitely wanted” a birth center birth (6). As such, birth centers are a high-value but under-utilized model of care, both for insurers and birthing people, where pregnant patients can receive midwifery services in a supportive homelike setting, delivering improved maternal and infant outcomes (7).

Despite being a promising model of care to improve perinatal health outcomes and reduce racial disparities, most states have few operational birth centers, with very limited geographic coverage. A review of literature identified a number of financial-related obstacles that birth centers face, including inadequate reimbursement for midwives and birth center services, inability to contract directly with managed care organizations, coverage limitations for services, limited ability to participate in delivery system reforms, state and local licensure laws, and malpractice premiums and certification costs (8–10). The national Strong Start for Mothers and Newborns II study testing enhanced prenatal care models for Medicaid beneficiaries found that many birth centers “struggled to serve Medicaid beneficiaries because reimbursement was inadequate to cover the baseline costs of care” (8). Although coverage for midwifery and birth centers is a federal Medicaid mandate under the Affordable Care Act, only 24% of birth center births nationally are among Medicaid participants, far below the 41% of births overall covered by Medicaid (7, 8, 11). Not all birth centers accept patients with Medicaid coverage, and among those that do, low Medicaid reimbursement rates are a primary barrier to birth center access and sustainability (8, 12).

A recent analysis for the National Academy for State Health Policy on midwifery reimbursement rates found a varied landscape of midwifery policies, with some states trying to expand the types and scopes of midwives permitted to practice and linking higher reimbursement rates to better perinatal access (13). However, little is known about how state Medicaid reimbursement rates vary for birth center facility fees besides wide variation and low rates (14). The objective of this study, therefore, was to examine variation in state Medicaid reimbursement rate policy for birth center facility fees, and best practices in rate-setting among comparable states. We anchored our analysis on New Jersey, identifying a sample of states with comparable policy, demographic, cultural and infrastructure characteristics.

## Material and methods

This study utilized a cross-sectional policy analysis by identifying state regulations or policy documents that provide Medicaid reimbursement rates for birth centers in select states. Due to the progressive maternal health policy environment in New Jersey, we sought documentation on Medicaid reimbursement rates for New Jersey and states with similar characteristics. New Jersey launched the NurtureNJ Strategic Plan in 2021, with bold practice and policy recommendations aimed at making New Jersey the safest and most equitable state in the U.S. to birth and raise a baby (15).

Two different state similarity methodologies were used to identify states with comparable policy or population characteristics. Bricker and LaCombe's method generates a state similarity network based on perceived similarity and confirms those with state policy adoptions (16). Jones's state similarity index incorporates five equally weighted categories of state characteristics (demographics, culture, politics, infrastructure, and geography) (17). The resulting similar states will vary depending on the anchor state, so using New Jersey as the primary state, these methods converged on eight similar states: Connecticut, Delaware, Illinois, Maryland, Massachusetts, New York, Pennsylvania, and Rhode Island. Policy searches in the nine-state sample were performed in August 2024 for currently approved state policies or documentation on Medicaid reimbursement rates.

We performed numerous web searches, and also used Perplexity.ai, an artificial intelligence (AI) search tool to help identify regulations, bills, rate sheets, fee schedules, or any other documentation available for each state. Perplexity is an AI-based search tool that includes citations and sources in its results, unlike other AI search tools that respond conversationally but without links to source documentation. New research is being generated on academic use of AI search tools and its benefits and limitations (18, 19). Here, AI was only used to help identify source documentation. Search phrases included “[state name] state regulations for birth center reimbursement by Medicaid,” “what is the Medicaid facility fee for birth centers in [state name],” and “what is the reimbursement rate for birth center services in [state name].” Variations on the search string provided slightly varying results. AI generated results were reviewed by study researchers.

A database was generated compiling state regulations and other documentation identifying the Medicaid facility fee rates for each state, along with relevant notes about coverage limitations. The primary outcome of interest was the published Medicaid reimbursement rate for birth center facilities in each of the nine states as of August 2024. Data sources for Medicaid facility reimbursement rates include the Connecticut Department of Social Services Provider Fee Schedule (20), Illinois Department of Healthcare & Family Service Birth Center Fee Schedule (21), Code of Maryland Regulations 10.09.85.06 (22), 101 Code of Massachusetts Regulations 355.00 (23), New Jersey Administrative Code 10:58-1.7 (24), and the Pennsylvania Department of Public Welfare Bulletin Volume 43 Issue 28 (25). Secondary outcomes were policies on reduced rates for hospital transfers, inclusion of newborn care reimbursement, and inclusion of midwifery or other professional services in the published rates. Transfer rates were noted in all available policies, but the other secondary outcomes were not regularly included in documentation and so not reported here. Related demographic data and birth center birth rates were also tracked to show the racial and economic context of each state, some of which are known drivers of maternal health disparities in the U.S.; data sources included MacDorman and Declercq and Kaiser Family Foundation (26–28).

Limitations of this research include the limited state sample. While 21 additional states cover birth center services with Medicaid dollars, not all publish their Medicaid reimbursement rates, and differences in policy environment, demographics and geography make comparison less relevant to our research objectives (29). But the methodological approach of using converging state similarity methods is repeatable and applicable to other state groupings. Another limitation is the lack of publicly-available Medicaid rates in many states, making national analysis and comparison difficult and incomplete. State regulations around birth centers are in flux as of 2025, and a more complete analysis of all state regulations and reimbursement rates for birth centers is a recommended area of future research. Lastly, other policy evaluation components (e.g., rate changes over time, regulation development, policy implementation or impact) were not assessed in this study.

## Results

Of the nine states in the sample, six had publicly published rates set through legislation or other official state documentation (Connecticut, Illinois, Maryland, Massachusetts, New Jersey, Pennsylvania); the other three states (Delaware, New York, Rhode Island) had evidence of Medicaid reimbursement for birth centers but no publicly available rate. The three states without published rates do have birth center licensing or regulations, and New York has Managed Care Organization reimbursement only, with no available state Medicaid payment (personal communication, February 2025). Table 1 provides a summary of characteristics and findings for the nine states.

**Table 1:** Birth center utilization and medicaid-reimbursed facility fees in a nine-state sample.

State	Births financed by medicaid *n* (%)	Births to Black mothers *n* (%)	Birth center births *n* (%)	Maximum medicaid-reimbursed facility fee	Facility fee reimbursement change for transfer to hospital	Date of last update
CT	13.425 (38)	4.298 (12.2)	104 (0.30)	$2,500	$1,000	2/22/23
DE	4.533 (42)	2.853 (25.9)	140 (1.29)	Not found	Not found	N/A
IL	49.758 (39)	19.296 (15.7)	<20	$2,544	Rate reduced to 15% plus $53.56/hr for “observation” (labor)	7/1/22
MD	27.584 (40)	20.348 (29.9)	326 (0.46)	$2,500	Reduced rate based on length of time laboring in birth center	4/6/21
MA	21.318 (31)	7.125 (9.6)	192 (0.27)	$6,012	Reduced rate for transfer based on “individual consideration”	1/19/24
NJ	31.012 (30)	12.911 (12.6)	<20	$1,300	$500 max.	6/4/18
NY	98.396 (47)	27.935 (13.7)	173 (0.08)	N/A	N/A	N/A
PA	44.317 (34)	16.616 (12.6)	1.711 (1.24)	$1,328	$628 max.	7/13/13
RI	4.177 (41)	842 (8.0)	<20	Not found	Not found	N/A

Among the nine states, New Jersey had the lowest rate of Medicaid-financed births in 2022 at 30%, followed by Massachusetts at 31%, Connecticut with 38%, and New York and Delaware had the highest rates of Medicaid-financed births at 47% and 42%, respectively. Delaware and Pennsylvania had the largest numbers of birth center births, but still a very small percentage of births overall (1.29% and 1.24%, respectively). Birth center utilization overall is relatively low in the sample of states; the birth center birth rate was less than 0.50% for all states except Delaware and Pennsylvania (1.29% and 1.24% respectively, in 2017) (26).

The maximum allowable Medicaid reimbursement rate for birth center facilities in the states with published rates was the lowest in New Jersey at $1,300, compared to $1,328 (PA), $2,500 (CT and MD), $2,544 (IL), and $6,012 (MA). If a hospital transfer occurs at any time after admission for labor, New Jersey's reimbursement rate drops to $500; Pennsylvania's is reduced to $628, Connecticut reimburses $1,000, Maryland's rate is modified relative to the duration of time spent at the birth center, the rate in Illinois is reduced to $381 (plus an additional hourly rate for labor “observation”), and Massachusetts's is modified on an individual basis, with no further details available. Additional Medicaid-reimbursable services are sometimes allowable beyond the facility fee, including additional provider fees, medication administration, and other services, but these were not a focus of the research.

## Discussion

This cross-sectional policy analysis of Medicaid reimbursement rates for birth centers produced two key findings. First, New Jersey and Pennsylvania's facility fee reimbursement rates are notably two to four-and-a-half times lower than comparable states, with New Jersey's being the lowest of states in this sample. These two states' rates are also the oldest, set in 2018 and 2013 respectively. The other four states with higher reimbursement rates have been updated since 2021, an indication of regular rate assessment, although the frequency of reassessment was unclear. States set Medicaid reimbursement rates according to different processes. Some, like Maryland, Massachusetts, and New Jersey are legislated in state code, which can require a lengthy legislative process for amendment. Other states (e.g., Illinois) establish regulatory authority and then update rates through an annual rate memo/bulletin or other periodic fee schedule process.

Second, we also found that many states had large reductions in reimbursement rates for hospital transfers. Approximately 20%–30% of birth center patients require hospital transfer before, during, or after birth, but facility fees are often “paid based on where the baby emerges, not where resources were expended during labor” (30). A reduced rate for shorter facility utilization, aligned with standard fee-for-service practices, may disincentivize birth centers from admitting patients they deem a risk of transfer. Although fee-for-service reimbursement models reward more intervention and services, value-based payment models may be more appropriate for birth care, where less intervention and more time are often required (31). When transfers are necessary, a value-based payment that rewards both the birth center and hospital for a successful outcome could be much more effective than splitting payments—and simultaneously improve collaboration and integration of health providers and systems. New Jersey's Medicaid program, NJ FamilyCare, is currently piloting a three-year Perinatal Episode of Care program that runs from 2022 to 2025 and is testing comprehensive clinical responsibility for perinatal outcomes (32). The pilot is clinician based, so could incorporate midwives and collaborating physicians serving birth centers and their collaborating hospitals; the pending results can inform future value-based payment policy.

It remains to be seen how increased rates alone improve actual utilization of birth centers. Of the state sample included in this analysis, the states with the highest Medicaid rates were not necessarily the states with the highest birth center birth rates. One hypothesis for this is related to timing of rate increase as opposed to actual reimbursement value. The states with higher rates (i.e., Connecticut, Illinois, Maryland, Massachusetts) have had more recent increases, three since the start of COVID and perhaps in response to increased demand for out-of-hospital birth during COVID. But the data for birth center births are lagged from 2017 (the most recent data available), so cannot be used to identify impact of the Medicaid policy updates (26).

According to a March of Dimes report on maternity care deserts, access to maternity care overall is relatively high in the nine sample states, averaging 87% of counties with full access, defined as counties with at least two hospitals and birth centers offering obstetric care, or more than 60 obstetric providers per 10,000 births (33). Higher maternity care access overall in the state sample may also correlate with the relatively low number of birth center births in these states. Research also indicates that site of delivery accounts for almost half of racial disparities in severe maternal morbidity rates between Black and White mothers, with Black mothers more likely to birth at high-risk hospitals for severe maternal morbidity (34, 35). In their study of births in 40 New York City hospitals, Howell and colleagues found that if Black mothers delivered in the same hospitals as White women, there would be almost 1,000 fewer severe morbid events. Better patient choice and access, as well as improved clinical protocols, team building, and improved communication within hospitals can improve outcomes (35). Birth centers with well-integrated midwifery-led teams could provide alternatives to high-risk hospitals for low-risk birthing people in these areas, as well as in maternity care deserts.

## Implications for practice and/or policy

These findings can help states and other jurisdictions improve funding structures for birth centers, improving access and practice sustainability. Regarding applicability of these findings to other states, demographic, cultural and geographic variations are important considerations. New Jersey, for example, is a very dense, relatively urban state with few rural regions with limited maternity care access. Only one of New Jersey's 26 counties (Cumberland) is defined as low access to maternity care, as compared to almost half of counties in all other states (33). States with vast rural or frontier regions tend to have better birth center access out of necessity, such as Alaska, Washington, Idaho, Oregon and Montana (26). Barriers to access in the state sample, such as limited patient demand and low reimbursement rates, are less likely in more rural states. Indeed, the Medicaid reimbursement rate in Alaska for birth center facility fees is set at 75% of the weighted average of corresponding hospital fees and updated annually, with a calculated rate of $2,888 in 2023—more than twice the reimbursement rate in New Jersey (36). In Minnesota, where 19.5% of counties are considered maternity care deserts and the birth center birth rate is 0.79% of births (26), birth centers are reimbursed at 70% of the hospital facility rate. Regulators there are weighing different rate setting methodologies to propose updated facility fee reimbursement rates in an upcoming legislative session (personal communication, August 2024).

Increasing Medicaid rates or switching to value-based payments for birth centers are potentially powerful policy solutions to improve access for diverse socioeconomic and racial/ethnic populations. As Medicaid reimbursement rates increase, commercial insurance tends to follow, which could further improve birth center sustainability through payor diversification, potentially allowing birth centers to take on more pregnant patients with Medicaid coverage or uninsured patients. Other regulatory pathways include expanding access to midwifery care through increased Medicaid reimbursement and reimbursing doula care to support patient knowledge and advocacy (13, 37).

## Conclusions

This nine-state analysis of birth center Medicaid reimbursement rates identified published facility fee rates for six states, ranging from $1,300 in New Jersey to 4.5 times that in Massachusetts. Hospital transfers result in even lower reimbursement rates and disincentivize an integrated system of care. The study anchored on New Jersey, and focused on states with similar population and policy characteristics including also Connecticut, Delaware, Illinois, Maryland, Massachusetts, New York, Pennsylvania, and Rhode Island. As such, results indicate a heterogeneous and unsupportive policy environment for birth centers in some states. Increasing Medicaid reimbursement rates for birth centers and using a value-based payment model could enhance birth center integration into the broader health ecosystem, improving access for low-risk women, especially Black and Hispanic women in the U.S, and set a better standard for other states aiming to improve access to this under-utilized care setting. Along with corresponding state efforts to improve access to midwifery care, these combined improvements in Medicaid reimbursement can increase birth center access and improve experience and outcomes among low-income pregnant people.

## Data Availability

The original contributions presented in the study are included in the article/Supplementary Material, further inquiries can be directed to the corresponding author.
